# Seasonal variations of triterpene acid contents in *Viscum album* L. on typical host trees of Hyrcanian forests

**DOI:** 10.1038/s41598-023-38649-x

**Published:** 2023-07-18

**Authors:** Arina Soursouri, Seyed Mohsen Hosseini, Farnoosh Fattahi

**Affiliations:** 1 Department of Forest Sciences and Engineering, Faculty of Natural Resources and Marine Sciences, Tarbiat Modares University, Noor, Iran; 2 Faculty of Natural Resources and Marine Sciences, Tarbiat Modares University, Noor, Iran

**Keywords:** Biochemistry, Cancer, Ecology, Plant sciences

## Abstract

*Viscum album* L. (mistletoe) is a semiparasitic plant of the Santalaceae family. A valuable group of bioactive compounds in mistletoe are triterpene acids (TTAs), which possess anti-inflammatory and anticancer properties. *Parrotia persica* and *Carpinus betulus* are the most common hosts of mistletoe in the Hyrcanian forests of Iran. This study was performed to compare the content of oleanolic acid (OA), betulinic acid (BA), and ursolic acid (UA) in the mistletoe foliage (stems and leaves) from *P. persica* and *C. betulus* in various seasons for the first time. The results showed that OA was the prevailing TTA in all samples, while UA was found in none of them. The maximum amount of OA (12.38 mg/g dry weight [DW]) and BA (1.68 mg/g DW) was detected in *V. album* from *P. persica* in summer. The minimum amount of OA (5.58 mg/g DW) and BA (0.72 mg/g DW) was observed in that growing on* C. betulus* in winter. However, the mistletoe from *C. betulus* showed the greatest level of OA in spring (9.06 mg/g DW) and BA in summer and autumn (0.92 and 0.97 mg/g DW, respectively). The data collected in this study complement existing research on this subject from around the world.

## Introduction

Parasitic interactions play an important role in all ecosystems and are very diverse among different organisms. These interactions encompass all groups of organisms, including phages, viruses, bacteria, animals (mostly insects), fungi, and flowering plants. Mistletoes are a well-known group of parasitic flowering plants^1^. *Viscum album* L., commonly known as European mistletoe, belongs to the Santalaceae family and is a semiparasitic evergreen species that inhabits a wide range of woody plants^2,3^. Mistletoe has a low photosynthetic rate and absorbs water and nutrients from host trees through its endophytic haustorium system. This semiparasitic plant is a threat to the survival of the host tree. Infested host trees may demonstrate water stress, deficiency of macro and microelements, loss of some carbohydrates, growth delay, and morphological abnormalities. Mistletoes cause low vigor in host trees and prepare them to be affected by attacks of other agents like insects or rottenness fungi^1,4^. However, *Viscum album* is an amazing medicinal plant with considerable pharmacological effects. The extracts of this plant are used as adjuvant cancer therapy^5,6^. Several studies have shown other biological effects of mistletoe, such as a remedy for cardiac diseases^7^, anti-diabetic potential^8^, apoptosis induction in acute lymphoblastic and myeloid leukemia^9,10^, as well as control of hypertension^11^. The medicinal activities of mistletoe are associated with several biologically active compounds such as viscotoxins, lipids, polysaccharides, flavonoids, alkaloids, and various (glyco) proteins^5,10,12^.

A main group of bioactive constituents of mistletoe is the pentacyclic family of triterpene acids (TTAs), like oleanolic acid (OA), betulinic acid (BA), and ursolic acid (UA) (C_30_H_48_O_3_)^13,14^. These mentioned compounds are reputed for their cytotoxic and antitumor activities, but due to their low solubility, they are not present at significant concentrations in aqueous extracts^9,10^.

The Hyrcanian forests, with a major area of about 1.9 million hectares in northern Iran, are located on the southern coasts of the Caspian Sea. This unique site contains valuable species such as *Parrotia persica*, *Populus caspica*, *Gleditsia caspica, Pterocarya fraxinifolia*, *Carpinus betulus*, etc.^15^. Two of the most abundant mistletoe host species in Iranian Hyrcanian forests are Persian ironwood (*P. persica* C.A. Mey.), an endemic deciduous tree from the Hamamelidaceae family^16^ and Hornbeam (*C. betulus* L.), a native deciduous tree belonging to the Betulaceae family^17^.

According to various studies, the active ingredients of mistletoe and its therapeutic effects can significantly change relating to the host species, harvest season, and growing conditions such as temperature and climate^14,18–23^.

Regarding the effect of harvest time, *V. album* plants from various deciduous host trees harvested at different seasons in Poland indicated the highest concentrations of TTAs in summer. Also, OA was the most abundant TTA in their extracts^18^. Likewise, Turker et al.^23^ reported that water and ethanol extracts of *V. album* plants inhabiting different host trees revealed various antitumor activities according to the host species. In their study, the water extract of *V. album* from cherry (*Prunus divaricate*) and wild pear (*Pyrus elaeagnifolia*), as well as the ethanol extract of mistletoe samples on pine (*Pinus sylvestris*) showed the highest antitumor effect, respectively^23^.

To the best of our literature review, there is no report on the TTAs content of Iranian mistletoe under the effects of host species and harvesting season. Because the content of bioactive compounds in mistletoe can vary depending on the host species and harvesting time, this study was conducted for the first time to evaluate the variations of TTAs content in Iranian *V. album* from* P. persica* and *C. betulus* in the Hyrcanian forests of Iran during different seasons.

## Results

As mentioned, to study the effect of two host species, *P. persica* and *C. betulus*, on the OA, BA, and UA content of mistletoe, the samples were harvested in summer (early September), autumn (early December), winter (early March), and spring (early June). The extract analysis indicated that OA and BA existed in all samples, but no UA was found.

### Effect of host species, *P. persica* and *C. betulus*, on the OA and BA content of mistletoe

**Figure 1:**
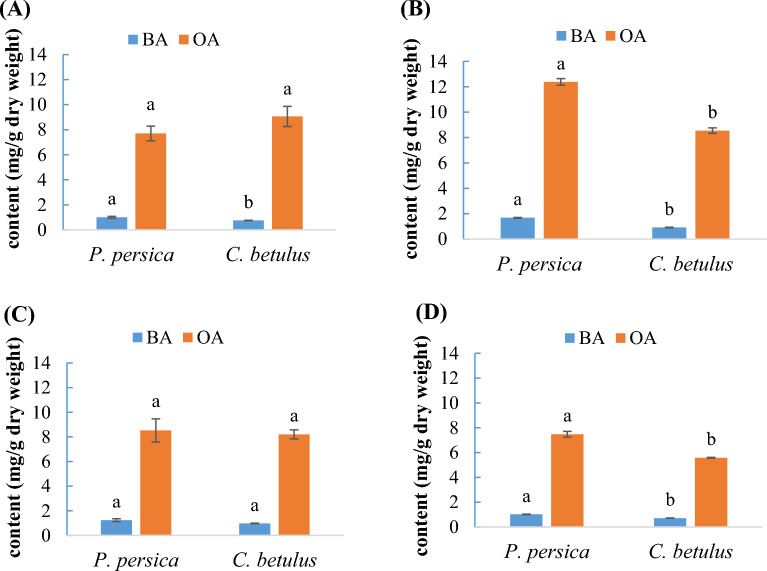
Effect of *P. persica* and *C. betulus* host species on oleanolic acid (OA) and betulinic acid (BA) content of *V. album* in (**A**) spring, (**B**) summer, (**C**) autumn, and (**D**) winter. Each value is the mean of three replicates. Different letters denote significant differences at P ≤ 0.01 with Duncan’s test.

According to Fig. 1, the mistletoe OA content was significantly affected by host species in summer and winter, so that the mistletoe growing on *P. persica* indicated the maximum concentration of OA in both seasons, summer (12.38 mg/g DW) and winter (7.48 mg/g DW) (Fig. 1B,D). It is noticeable that the level of OA in mistletoe from *C. betulus* was somewhat higher than that in mistletoe from *P. persica* only in spring, when they were 9.06 mg/g DW and 7.71 mg/g DW, respectively.

Betulinic acid was significantly affected by host species in all seasons except autumn. Mistletoe growing on *P. persica* produced the highest content of BA in three seasons, including spring, summer, and winter, with concentrations of 1.00 mg/g DW, 1.68 mg/g DW, and 1.02 mg/g DW, respectively (Fig. 1A,B,D).

### The seasonal variations of OA and BA content in mistletoes from different host species

The analysis of variance (ANOVA) indicated that the type of host species, season, and their interaction had a significant effect on the OA and BA content of mistletoe at the 1% probability level (Table 1).

**Table 1:** Analysis of variance of OA and BA contents in the mistletoes growing on *P. persica* and *C. betulus* in different seasons.

Sources of variation	Degree of freedom	Mean square	
		BA	OA
Host species	1	0.930**	8.263**
Season	3	0.257**	15.515**
Host species × Season	3	0.093**	7.355**
Error	16	0.008**	0.768**

**Significant difference at 1% probability level.

**Figure 2:**
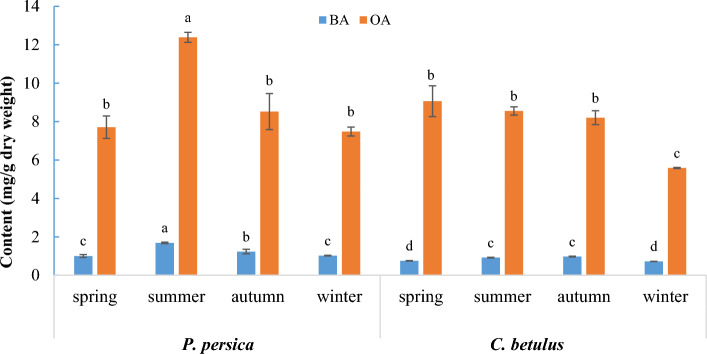
Comparing the seasonal variations of oleanolic acid (OA) and betulinic acid (BA) content in *V. album* growing on *P. persica* and *C. betulus*. Each value is the mean of three replicates. Different letters denote significant differences at P ≤ 0.01 with Duncan’s test.

As shown in Fig. 2, OA was the prevailing TTA in mistletoe samples from both host species in all seasons*.* The quantity of OA ranged from 5.58 to 12.38 mg/g DW, which was approximately 7.5-fold higher than the amount of BA, which varied from 0.72 to 1.68 mg/g DW.

The maximum concentration of OA (12.38 mg/g DW) and BA (1.68 mg/g DW) was found in the mistletoe from *P. persica* species in summer, while the lowest content of OA (5.58 mg/g DW) and BA (0.72 mg/g DW) was detected in the mistletoe growing on *C. betulus* in winter (Fig. 2).

In the mistletoe from *P. persica*, there was no significant difference in OA quantities between spring, autumn, and winter samples, although the lowest amount of that was detected in winter (7.48 mg/g DW). Also, the minimum concentration of BA in these samples was observed in spring and winter (1.00 and 1.02 mg/g DW, respectively) (Fig. 2).

The TTAs analysis of the mistletoe growing on *C. betulus* during four seasons showed that there was no significant difference in the OA values in the spring, summer, and autumn samples; nevertheless, OA concentration was at the highest level in spring (9.06 mg/g DW) (Fig. 2). As well, the highest content of BA in the mistletoe from *C. betulus* was detected in summer and autumn (0.92 and 0.97 mg/g DW, respectively) (Fig. 2).

Overall, as shown in Table 2, the mistletoe growing on *P. persica* had the maximum content of total OA (36.09 mg/g DW), total BA (4.94 mg/g DW), and therefore total OA + BA (41.02 mg/g DW) of four seasons. However, the highest ratio of OA to BA (9.34) was detected in that growing on *C. betulus*, which was 1.3-fold higher than in the mistletoe on *P. persica*. (Table 2).

**Table 2:** Comparing the total value of OA, BA, OA + BA, and their ratio (OA/BA) in *V. album* from *P. persica* and *C. betulus* during four seasons.

Host species	Total OA^ns^ (mg/g DW)	Total BA** (mg/g DW)	Total OA + BA* (mg/g DW)	OA/BA ratio**
*P. persica*	36.09 ± 1.51^a^	4.94 ± 0.11^a^	41.02 ± 1.62^a^	7.30 ± 0.14^b^
*C. betulus*	31.39 ± 1.08^a^	3.36 ± 0.04^b^	34.75 ± 1.10^b^	9.34 ± 0.25^a^

Each value is a mean ± SE (n = 3). Different letters denote significant differences at *P ≤ 0.05, **P ≤ 0.01, and ^ns^non-significant using Duncan’s test.

Moreover, the total average concentration of each TTA, extracted from mistletoes on both host species, varied in different seasons as follows: 8.38 (spring), 10.47 (summer), 8.36 (autumn), and 6.53 (winter) mg/g DW for OA, as well as 0.88 (spring), 1.3 (summer), 1.1 (autumn), and 0.87 (winter) mg/g DW for BA. These fluctuations of OA and BA levels revealed that TTAs accumulation gradually increased from winter to spring, peaked in summer, then decreased to autumn, and subsequently reached a minimum concentration in winter.

## Discussion

Triterpene acids, OA, BA, and UA, are a group of important bioactive compounds in *V. album* L., which are famous due to their apoptosis induction in cancer cell lines and cytotoxic anti-cancer property^10,13^. *Viscum album* L. is an evergreen semi-parasitic plant, and its phytochemical components can be affected by the host species and its vegetative period^13,18^. As described before, in the current study, the effect of *P. persica* and *C. betulus* host species was assessed on the quality and quantity of Iranian *V. album* TTAs at different harvest times.

The findings of this investigation revealed that the quality and type of TTAs in mistletoe samples from both host species during different seasons were identical. All samples included OA and BA, while OA was the main TTA, but UA was not discovered in any of them. This trend of TTAs composition was in agreement with the Wójciak-Kosior et al.^18^ report, which, to the best of our knowledge, is the only study about the variations of TTAs content in European mistletoes growing on several host species in different seasons. However, research on other bioactive compounds of *V. album* also showed that host species and harvest time had no effect on the type of viscotoxin compounds in *V. album* subsp. *album*^24^ and components of flavonoid glycosides in *V. album* var. *coloratum*^25^, but the quantity of these compounds varied depending on the host species. Likewise, the present results indicated a significant variation in the content of TTAs based on the type of host species and also seasonal alterations. Regarding the effect of host species, our results indicated the mistletoe from *P. persica* contained higher levels of OA and BA in almost all seasons, with the only exception of spring, when its OA content was lower than that in the mistletoe from *C. betulus*. Furthermore, the total average concentration of TTAs varied from the lowest level in winter to a higher level in spring, reached a maximum point in summer, and gradually decreased until autumn.

Therefore, the highest concentration of OA and BA was found in the mistletoe from *P. persica* in summer, while the minimum content of both compounds was observed in samples from *C. betulus* in winter. Similarly, Wójciak-Kosior et al.^18^ confirmed the host-dependent OA and BA accumulation in *V. album* growing on deciduous Angiospermae host species, with the utmost concentration in summer. Yet, regarding *V. album* growing on *Pinus sylvestris* (Gymnospermae), they found the highest concentration of OA and BA in winter.

Mistletoe is a semi-parasitic plant that absorbs water and nutrients from host species. As a consequence, variations in mistletoe bioactive compounds under the effect of host species are expected^5^ and have already been confirmed for other phytochemicals of *V. album,* such as phenolic compounds^26^, viscotoxins, and lectins^5^, as well as some small molecules as important biomarkers^6^. Also, different seasons can influence the mistletoe biochemical compounds through environmental factors or particular vegetation periods^20,27^. In this regard, Barbasz et al.^27^ reported significant changes in concentrations of some other mistletoe compounds like sugars, lipids, and polyamines in different periods of the year. Likewise, Vicaş et al.^20^ found variations in total phenolic content and antioxidant activities of *V. album* depending on the harvest times.

Several studies have shown that medicinal plants contain the highest content of secondary metabolites at the full flowering stage^28–31^. Mistletoe flowering time is during the winter months, from February to March, until April^32^, which coincides with dormancy and the absence of leaves in deciduous host species. Whereas in the present study and the Wójciak-Kosior et al.^18^ experiment, the highest level of TTAs in *V. album* from deciduous host trees was found in the summer. According to this finding, it seems that mistletoe TTA biosynthesis is more dependent on the growth periods of the host species.

Triterpenes are one of the largest and most varied plant bioactive compounds, derived from a linear 30-carbon precursor, 2,3-oxidosqualene^33^. The production of pharmaceutical terpenoids in medicinal plants can be improved by regulating the primary metabolism. Carbohydrates are the main metabolites of primary metabolism, and their synthesis is affected by the photosynthesis rate^34^. Thus, the production of terpenoids in plants is directly linked to photosynthesis and, consequently, the biosynthesis of related precursor metabolites^35^. Zhang et al.^35^ demonstrated that the increase in the photosynthetic capacity of *Glechoma longituba* leaves under the influence of blue and green lights enhanced TTAs accumulation in this plant. Also, other studies indicated the crucial role of carbohydrates as precursor metabolites in the biosynthesis of plant terpenoid compounds. In this regard, feeding *Euphorbia lathyris *seedlings with various precursor compounds showed that sucrose and, to a lower extent, glucose are the main metabolites in the latex triterpene synthesis of the seedlings and produced the highest amount of triterpenes per seedling per day^36^. Likewise, the addition of higher concentrations of sucrose in cell suspension cultures of *Uncaria tomentosa*^37^ and *Salvia fruticosa*^38^ led to an increase in TTAs contents. This positive effect of sucrose on TTA accumulation could be attributed to the chemical structure of these secondary metabolites^38^.

Mistletoe, in addition to water and mineral salts, receives 22.6 to 45% of its carbon from the host species^39^. The transfer of carbohydrate from the host to the mistletoe considerably depends on the carbohydrate content of the host xylem sap^40^. Escher et al.^40^ have shown that the seasonal variations of carbohydrate concentration and composition in the xylem sap of *V. album* are similar to their patterns in the respective host tree xylem sap. In their experiment, the carbohydrate content of *V. album* on a deciduous host tree, *Populus* × *euamericana*, changed from the highest concentration in spring to a low concentration in summer and autumn^40^.

Moreover, mistletoe is a partially heterotrophic plant that appears to be able to meet some of its carbon demand through a high transpiration rate. However, the transpiration rate of mistletoe on deciduous trees reduces during the leafless period of the host tree^32^.

In the present study, the concentration of OA and BA in mistletoe from *P. persica* gradually increased in spring and reached a significant peak in summer. However, in samples from *C. betulus,* a significant increment of OA content was detected in spring, summer, and autumn, respectively, whereas the highest level of BA was observed in summer and autumn. It seems that the enhancement of mistletoe TTAs content is synchronous with the beginning of active growth of the host tree in spring, when the concentration of carbohydrates increases in the host and subsequently in the mistletoe. Carbohydrate accumulation as a precursor primary metabolite for TTAs biosynthesis may cause an increase in mistletoe TTAs content in spring and summer. In correspondence with our assumption, Lázaro-González et al.^41^ demonstrated that *Viscum album* subsp. *austriacum* obtains metabolites mostly through the primary metabolism of the host tree and produces its defensive chemicals. Besides, in agreement with our findings, they reported a higher concentration of a few amino acids, carbohydrates, and defense compounds of *V. album* subsp. *austriacum* in the summer^41^.

From another point of view, the increase of secondary metabolite production in plants is a response to stress conditions such as high temperatures^42^. As a result, the increase in temperature and daylight length in spring and especially summer can stimulate mistletoe defense responses, leading to a rise in TTAs levels during these seasons. This hypothesis is in line with Alqahtani et al.^43^ findings on triterpene accumulation in *Centella asiatica*, which increased in warm climates with long daylight hours in summer.

Taken as a whole, it seems that the improved primary metabolite content of mistletoe, along with environmental stresses during spring and summer, caused the highest TTAs content in the summer mistletoe samples, but further experiments are required to provide more accurate explanations for this subject.

## Materials and methods

### Plant material

**Figure 3:**
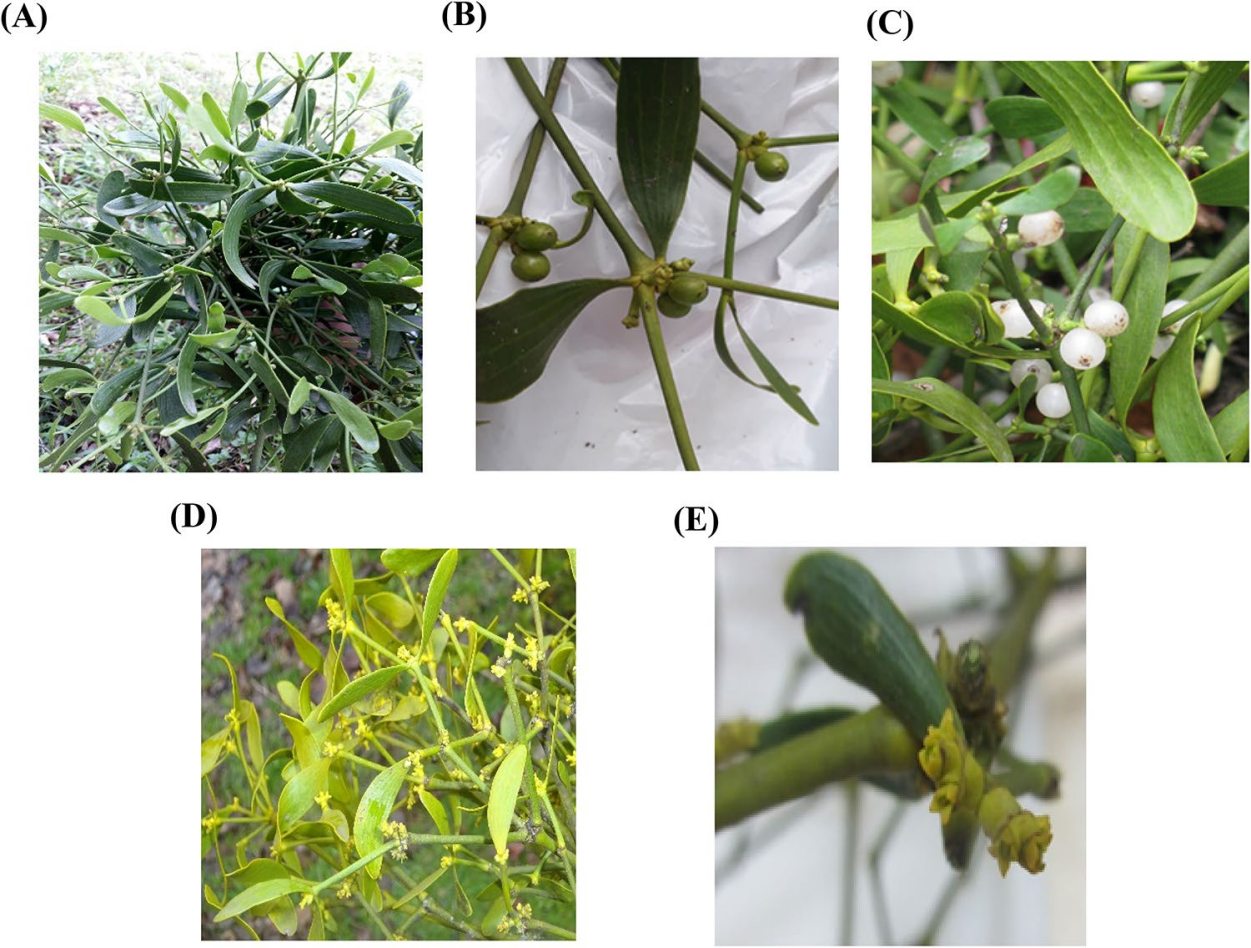
*Viscum album* in different seasons (**A**) spring; (**B**) summer; (**C**) autumn; (**D**) winter; (**E**) female flower in winter.

**Figure 4:**
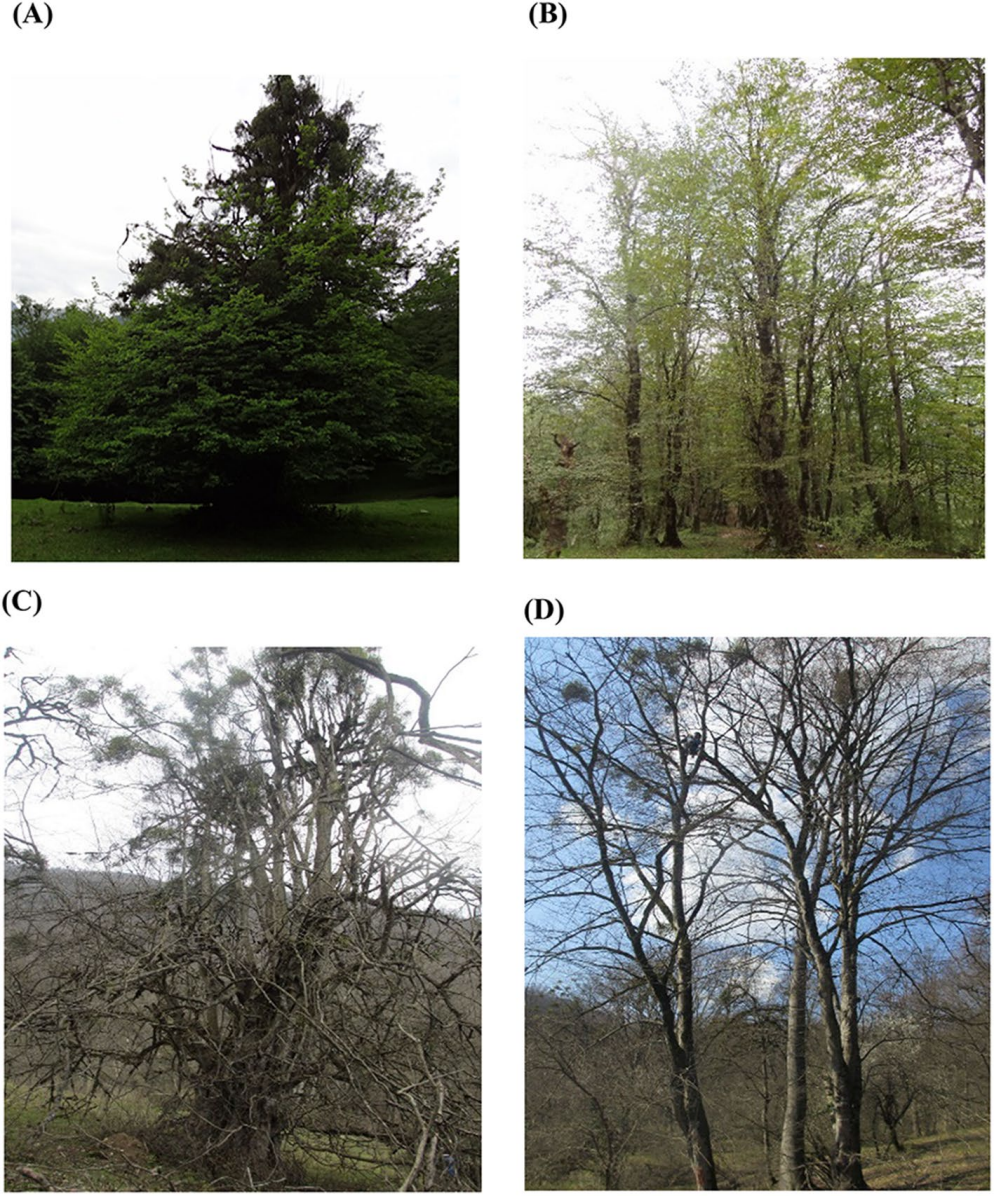
Two host species (**A**) *P. persica* in spring; (**B**) *C. betulus* in spring; (**C**)* P. persica* in winter; (**D**)* C. betulus* in winter.

The collection of *V. album* samples for this study was fulfilled according to legislation and the formal permission of the Natural Resources and Watershed Management Organization of Iran. *Viscum album* specimens were collected as wild plants from *P. persica* and *C. betulus* host species in the Daeiz area of the Kelerd forest of Hyrcanian forests in Mazandaran province of Iran during the autumn (early December) in 2017, winter (early March), spring (early June), and summer (early September) in 2018 (Figs. 3 and 4). A voucher specimen of *V. album* (No. 1100) has been deposited in the Herbarium of Yasouj University, Iran.

The altitude gradient of the study area is 700–1100 m a.s.l. The region has a semi-humid climate, with a mean annual temperature of 17 °C and a mean annual precipitation of 668 mm.

The host plants were selected in such a way as to have homogeneous ecological conditions as well as the same diameter (at breast height, DBH), height, and morphology without other stresses such as pests, diseases, etc. For sampling, five branches of the last three generations of* V. album* foliage (stems and leaves) were harvested from each mistletoe on three individuals of each host species during four seasons. At the harvest times, the mistletoe was in full flowering (winter), beginning of fruiting (spring), green berry (summer), and ripe white berry (autumn) phenological stages (Fig. 3). Mistletoe samples, after being placed in a liquid nitrogen tank for a few seconds, were stored at − 80 °C until extraction. All methods were carried out under the relevant guidelines in the method section.

### Ultrasound assisted triterpene acid extraction

The plant samples were freeze-dried (OPERON, OPR- FDU- 7012, Korea) for 72 h and then powdered using a mill. One gram of plant powder with three replicates was dissolved in 25 ml of ethyl acetate and placed in an ultrasonic bath (ELMA, E120H, Germany) at 35 kHz frequency and 35 °C temperature for 30 min. The sonicated samples were centrifuged at 10,000 *rpm* for 10 min. The plant residue was extracted twice more with a portion of the new solvent.

The final volume of extract after filtering by a syringe head filter (0.45 µm) was concentrated by a rotary evaporator until the solvent completely disappeared. The residue was resolved in 10 ml of methanol and kept in a refrigerator at 4 °C^14,18^.

### HPLC analysis

Mistletoe TTAs, including OA, BA, and UA, were measured by an HPLC system (Waters 2695, USA) equipped with a Photodiode array (PDA) detector (Waters 996, USA) at a wavelength of 210 nm. Separation was carried out on a 25 cm × 4.6 mm Eurospher 100–5 C18 analytical column. The mobile phase in isocratic mode consisted of methanol–water-phosphoric acid (87:12.95:0.05, v/v/v) at a 1 ml/min flow rate. For each injection, 20 µL of the extract was used. The amount of TTAs, including OA, BA, and UA, was determined based on the calibration curves of the standard compounds in 50, 100, 150, and 250 μg/mL concentrations (Sigma Co.).

### Statistical analysis

First, the normality of data distribution was checked and confirmed using the Kolmogorov–Smirnov test (P > 0.05). Levene's test was also used to evaluate the assumption of data variance homogeneity.

The data were analyzed by one-way and two-way ANOVA to determine the significant levels. The mean values were compared using Duncan's test at the 5% probability level. All statistical analyses were performed by SPSS 23.0 software.

## Conclusion

The interaction between host species and parasites is very complex, and the role of host species in this interaction requires considerable research effort. This study was the first evaluation of the effect of host species and season on the TTAs content of *V. album* in the Hyrcanian forests of Iran. Iranian *V. album* from *P. persica* and *C. betulus* contained two kinds of TTAs, including OA and BA in all seasons, and OA was the dominant TTA. Ursolic acid was not found in any samples. Mistletoe from *P. persica* demonstrated the maximum concentration of OA and BA in summer, while mistletoe growing on *C. betulus* contained the highest amount of OA in spring and the highest content of BA in summer and autumn. Overall, to achieve the highest content of TTAs, harvesting the mistletoe growing on *P. persica* in summer was emphasized by the findings of this research.

## Data Availability

All data generated or analysed during this study are included in this published article.

## Abbreviations

BA: Betulinic acid
DW: Dry weight
OA: Oleanolic acid
TTAs: Triterpene acids
UA: Ursolic acid
